# Anti-IgLON5 Disease: A Case With 11-Year Clinical Course and Review of the Literature

**DOI:** 10.3389/fneur.2019.01056

**Published:** 2019-10-02

**Authors:** Mette Scheller Nissen, Morten Blaabjerg

**Affiliations:** ^1^Department of Neurology, Odense University Hospital, Odense, Denmark; ^2^Department of Clinical Research, University of Southern Denmark, Odense, Denmark; ^3^BRIDGE, Brain Research - Inter-Disciplinary Guided Excellence, University of Southern Denmark, Odense, Denmark

**Keywords:** autoimmune encephalitis, IgLON5, inflammation, tau, immunology

## Abstract

**Background:** Anti-IgLON5 disease is a novel disorder with a complex interplay between inflammation and neurodegeneration. Patients develop antibodies against IgLON5 but also deposition of neuronal tau protein. Symptoms often have an insidious onset, slow progression and mimic other neurological disorders. Here we report a case with severely prolonged 11-year disease course and provide a review of current reported cases with focus on presentation, work-up, treatment, and outcome.

**Method:** All reported cases of anti-IgLON5 disease were evaluated. Cases reported twice (in case series and as single case reports), were carefully excluded.

**Results:** Most patients display a characteristic sleep disorder with severe insomnia, non rapid eye movement (NREM) parasomnia, with finalistic movements and sleep disordered breathing (stridor and obstructive sleep apnea). Other symptoms are bulbar involvement, gait instability, movement disorders, oculomotor abnormalities, dysautonomia, and peripheral symptoms. Antibodies are present in both serum and CSF and there is a strong correlation with human leukocyte antigen (HLA) DRB1^*^10:01 and HLA-DQB1^*^05:01. Neuropathological examination reveals neurodegeneration with neuronal tau deposits in regions that correlate with the clinical presentation (e.g., predominantly hypothalamus and tegmentum of the brain stem). Majority of cases respond partially to immunotherapy. Cases, who received no treatment or treatment with IV corticosteroids alone, had a higher mortality than cases treated with more potent immunotherapy.

**Conclusion:** The clinical spectrum of Anti-IgLON5 disease continues to expand. Further studies are needed to elucidate the pathophysiology, therapeutic strategies and outcome in this novel disorder. Aggressive immunotherapy seems to increase survival.

## Background

The recently described disease with antibodies against immunoglobulin-like cell adhesion molecule 5 (IgLON5), is characterized by a distinctive sleep disorder associated with a broad variety of neurological symptoms such as gait instability, movement disorders, and brainstem involvement (1). Antibodies against IgLON5 have been described to cause irreversible internalization of surface IgLON5 and postmortem studies have demonstrated deposits of hyperphosphorylated tau (p-tau), with predominant involvement of hypothalamus and tegmentum of the brainstem, but also hippocampal formation and cerebellum (2, 3). Strong association with human leukocyte antigen (HLA) DRB1^*^10:01 and HLA-DQB1^*^05:01 alleles has been reported, making the IgLON5 disease a complex interaction between neurodegeneration and neuroimmunology with a genetic predisposition (4). Anti-IgLON5 disease differs from previous described autoimmune encephalitis (AIE) syndromes by a protracted clinical disease course, deposition of tau and a variable effect of immunotherapy, making it a challenge to diagnose and treat (4, 5). Since 2014, more than 60 cases have been reported, expanding the spectrum of neurological symptoms (4–19). Here we present a case report displaying a severely prolonged 11-year disease course and review of the current literature, focusing on clinical presentation, work-up, treatment and outcome of patients with anti-IgLON5 disease.

## Methods

All cases and case series were thoroughly examined. One case series, focusing on post-mortem findings, reported three cases previously described, and three cases with “probable” anti-IgLON5 disease due to neuropathological findings, but with unknown antibody status (3). These three probable cases, were not included in this review. One case series included previously published single cases and small case series (4). These duplicated cases were carefully excluded. We thus ended up with a review of 58 cases including our own.

The presented case provided informed consent for publication.

## Case

In January 2019, a 61-year old male, with a 1-year history of diagnosed obstructive sleep apnea (OSA), was admitted in a state of unconsciousness due to hypercapnia. He had an 11-year history of slowly progressive diplopia, hoarseness, slurred speech, dysphagia and sleep disturbances. At disease onset the initial symptoms were diplopia and mild dysphagia. On suspicion of multiple sclerosis a brain MRI was performed. It showed T2 weighted unspecific white matter (WM) hyperintensities in the brainstem. Cerebrospinal fluid (CSF) analysis revealed mild pleocytosis (15 white blood cells/uL), but normal protein levels and no oligoclonal bands (OCB). Visual and somatosensory evoked potential (VEP/SSEP) were normal. Multiple subsequent brain MRI's showed no further progression of the WM hyperintensities.

In 2018 he developed respiratory symptoms and was diagnosed with OSA (Apnea Hypopnea Index: 25). Despite continuous positive airway pressure (CPAP) treatment, he was admitted several times with respiratory failure at night within the last year, and his wife reported sleep abnormalities with atypical movements. Within the last 6 months symptoms progressed and he developed mild gait imbalance and behavioral changes with disinhibition.

Neurological examination revealed horizontal gaze palsy, ptosis of the left eyelid, mild dysarthria, oro-facio-mandibular dystonia, and mild tetraparesis with spasticity and Babinski's sign in his lower right extremity. Moreover, a mild gait ataxia and fasciculations on both upper and lower extremities were noticed.

Laryngoscopy was performed showing bilateral vocal cord palsy. Whole-body 18-FDG PET CT scan, electromyography (EMG), and nerve conduction (ENG) studies showed no abnormalities. Acetylcholine receptor antibodies were negative. A Mini Mental State Examination showed mild cognitive impairment (24/30) with visuospatial abnormalities.

CSF analysis was normal. Because of the slowly progressive symptoms including sleep disturbances, OSA, bulbar symptoms and gait imbalance, antibodies against IgLON5 were tested and found strongly positive in both serum and CSF (1:1,000 and 1:10 CBA Euroimmun) confirming the diagnosis. Analysis for IgG isotypes showed predominantly IgG4 isotype antibodies and the patient was HLA DRB1^*^10:01 and HLA-DQB1^*^05:01 positive (kindly performed by Dr. Gaig and colleagues).

Immunotherapy was initiated with high dose intravenous corticosteroids and therapeutic plasma exchange (TPE). Subsequent polysomnography (PSG) showed severe insomnia with overall 24 min of sleep, a sleep efficiency of 4%, stridor, vocalizations and abnormal movements both during wakefulness and sleep. Neither N3 sleep stages nor REM sleep was present. The patient and his spouse reported subjective improvement on gait imbalance and sleep, but no significant clinical improvement was found on neurologic examination. Second line treatment with Rituximab was initiated.

Three weeks later, he developed hypercapnia and respiratory failure during daytime, requiring mechanical ventilation. Repeated antibody analysis still showed strongly positive anti-IgLON5 antibodies in serum (1:100) but only mildly positive in CSF (1:1). Renewed TPE was initiated. At time of review the patient was fully awake, mRS score 2, but had undergone tracheotomy and received assisted mechanical ventilation at night. Sleep quality was improved and movements during sleep diminished.

## Demographics and Clinical Presentation

Anti-IgLON5 disease affects men and women equally and only 10% of patients have a history of autoimmune disease. In currently documented cases, median age at diagnosis was 62 years, ranging from 45 to 79 years. Only 11% had a history of malignancy, with no clear tendency of cancer type (Table 1).

**Table 1:** Demographics, antibody status, and CSF findings.

**Demographics**	**No. (%)**
Sex, female (*n = 64*)	32 (50)
Age at diagnosis (*n* = 35) (years, range)	62 (45–79)
Hx autoimmune disease (*n* = 58)	6 (10.3)
Hx of malignancy (*n* = 36)	4 (11.1)
**Antibody status CSF and serum**	**Positive**
CSF IgLON5 (*n* = 40)	38 (94.9)
Serum IgLON5 (*n* = 63)	63 (100)
IgG isotype, serum (*n* = 48)	
- IgG1	45 (93.8)
- IgG2	30 (62.5)
- IgG3	23 (47.9)
- IgG4	44 (91.7)
HLA-DRB1^*^10:01; DQB1^*^05:01 alleles (*n* = 26)	24 (92.3)
**CSF findings (*****n*** **= 41)**	**No. (%)**
Pleocytosis (>5 leukocytes/μL)	10 (24.4)
Increased protein (>45 mg/dL)	20 (48.8)
Oligoclonal bands (*n* = 29)	3 (10.3)
Tau (*n* = 6)	1 (16.7)^*^
P-tau (*n* = 7)	2 (28.6)^*^
β-amyloid (*n* = 5)	0^*^

* *From single case series included in Gaig et al. (4)*.

Symptoms are often heterogeneous, with insidious onset and slow progression. The median time from symptom onset to diagnosis was 12 months (range 3 weeks to 11 years—Supplementary Table 1).

The most prominent feature of the anti-IgLON5 disease is a distinctive sleep disorder, including both REM and non-REM parasomnia, finalistic movements and sleep disordered breathing with stridor and OSA (4, 5, 20). The NREM parasomnia seen in IgLON5 positive patients, differs from conventional NREM parasomnias (e.g., confusional arousals, sleep walking, sleep terrors), as it consists of poorly structured N2 sleep and undifferentiated NREM sleep (20). In cases, where detailed information was available, sleep disorder was present in 83% of patients and as part of initial symptoms in 40% (Table 2). Many patients report excessive daytime sleepiness and bed partners often complain of vocalizations, snoring and abnormal behavior and movement during sleep (20).

**Table 2:** Symptoms at onset vs. during disease and imaging findings.

**Symptom**	**Initial symptom (*n = 38*) No. (%)**	**Present overall (*n* = 58) No. (%)**
Sleep	15 (39.5)	48 (82.8)^*^
Obstructive sleep apnea	1 (2.6)	34 (58.6)
Bulbar dysfunction	8 (21.1)	44 (75.9)
Oculomotor dysfunction	1 (2.6)	25 (43.1)
Movement disorder	7 (18.4)	26 (44.8)
Gait disorder	10 (26.3)	37 (63.8)
Neuropsychiatric symptoms	3 (7.9)	29 (50)
Peripheral nervous system	1 (2.6)	12 (31.6)^**^
Autonomic dysfunction	0	24 (41.4)
**MRI (*****n*** **= 57)**	**No. (%)**	
Normal	46 (80.7)	
Abnormal	11 (19.3)	
- Brainstem atrophy	3	
- Bilat hippocampal atrophy	1	
- Cerebellar atrophy	2	
- Hypothalamic hyperintensities, T2	1	
- Focal enhancement of leptomeninges + edema, fronto-temporal enhancement	1	
- Brainstem hyperintensities, T2	1	
- Thalamic hyperintensity, T2	1	
**FDG PET CT (*****n*** **= 8)**	**No. (%)**	
- Normal	4 (50)	
- Abnormal	4 (50)	
- Striatal + brainstem hypermetabolism	1	
- hypermetabolism basal ganglia + cerebellum	1	
- Hypermetabolism left frontal, temporal lobe. bilat caudate nucleus and putamen	1	
- Hypermetabolism sensori-motor cortex, basalganglia, cerebellum	1	
**DAT-SPECT (*****n = 1*****)**	**No**.	
Normal	1	
**Tau-PET (*****n = 1*****)**	**No**.	
Increased tau brain stem and cerebellum	1	
**TSPO-PET (*****n = 1*****)**	**No**.	
Microglia activation in leptomeninges	1	

* *Five patients had unknown sleep pattern (not available), five patients without sleep disturbances*.

** *Calculated from n = 37*.

**Figure 1:**
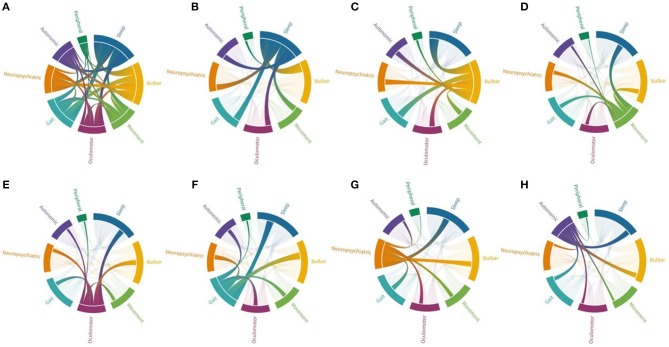
Connections between different symptom groups in anti-IgLON5 disease. Each symptom group is represented by a fragment of the outer circular layout. Arcs represent connection and flow between groups. Fragments and arch sizes are equal to percentage of overall symptoms/flow between symptom groups. **(A)** Shows the combination of all symptoms. Majority of cases experienced sleep disorder and bulbar symptoms, but almost all patients have three symptoms or more. **(B)** When focusing on patients with sleep disorder, most cases also display bulbar symptoms followed by gait impairment, while association with peripheral symptoms is the rarest combination. **(C)** Patients with bulbar symptoms mainly have sleep disorder, followed by gait impairment, oculomotor abnormalities and neuropsychiatric symptoms. **(D)** Patients with movement disorders (mainly chorea and parkinsonism) have associative sleep disorder and bulbar symptoms, but also gait impairment. **(E)** Oculomotor abnormalities were associated with sleep disorder, bulbar symptoms, and gait impairment. **(F)** Patients with gait impairment were also found to have mainly sleep disorder, bulbar symptoms, but also neuropsychiatric symptoms. **(G)** Neuropsychiatric symptoms were associated with mainly sleep disorder, bulbar symptoms, and gait impairment. **(H)** Dysautonomia was also associated to with these features.

Apart from sleep disturbances, the most frequent symptoms at disease presentation are gait instability (e.g., imbalance and ataxia), bulbar symptoms (dysphagia, dysarthria, laryngospasm, and respiratory symptoms) and movement disorders (chorea, parkinsonism, dystonia), accounting for >60% of symptoms (Table 2). Gait instability is often mild to moderate, and not always due to ataxia. Dysphagia is the most reported bulbar symptom (present in 67% of cases) and patients often report mild-moderate or intermittent problems swallowing. However, some patients experience severe dysphagia, with significant weight loss requiring a percutaneous endoscopic gastrotomy. Dysarthria (present in 38% of cases) is often characterized by hoarseness and dysphonia and accompanied by laryngospasms or vocal cord palsy. Additional often reported symptoms include oculomotor abnormalities in 43% (vertical and horizontal gaze palsy, nystagmus, ptosis, hypometric saccades), neuropsychiatric symptoms in 50% (cognitive impairment, memory deficits, and hallucinations), dysautonomia in 41% (urinary incontinence or urgency, constipation, anhidrosis), and symptoms of the peripheral nervous system in 32% (fasciculations, cramps, neuropathy) (Table 2). An overview of symptoms and their association is provided in Figure 1.

The heterogenous clinical presentation of neurological symptoms makes it difficult to distinguish anti-IgLON5 disease from other neurological diseases. In a previous report, four clinical phenotypes were proposed, (I) predominant sleep disorder, (II) bulbar syndrome, (III) gait instability and oculomotor involvement resembling Progressive Supranuclear Palsy (PSP-like syndrome), and (IV) cognitive impairment with/without chorea (4). Since then, case series and cases have described various phenotypes broadening the spectrum of anti-IgLON5 disease even further. Sudden onset with acute encephalic presentation and dyskinesia has been described in at least two cases (17, 18). One patient presented with severe muscle stiffness and hyperekplexia as seen in stiff-person syndrome (SPS) (5) and other case reports find fasciculations and peripheral symptoms such as peripheral nerve palsy, atrophy and muscle cramps (5, 9, 14, 16) (including our case). Depending on the presentation and combination of symptoms the clinical profile makes anti-IgLON5 disease easily misdiagnosed. The sleep disorder could be interpreted as pure OSA or REM sleep behavior disorder. A combination of sleep disorder, bulbar symptoms, gait imbalance, and movement disorders can resemble symptoms seen in neurodegenerative diseases such as PSP or multiple system atrophy (MSA). And a clinical profile dominated by respiratory failure, autonomic dysfunction, and hyperekplexia can be interpreted as neuromuscular or motor neuron diseases (MND).

Anti-IgLON5 disease is a rare disease, with an estimated incidence of 1/150,000 (5). Several studies have investigated subpopulations (e.g., sleep, neurodegenerative, and psychiatric cohorts) without finding a significant level of anti-IgLON5 positive cases (1, 21, 22). Thus, antibody testing in the absence of combination of abovementioned neurological symptoms is not recommendable.

The symptoms in anti-IgLON5 disease are likely explained by neuropathologic findings in post mortem studies (1, 3). The p-tau deposition in hypothalamic and hippocampal areas explains the characteristic sleep disorder and amnestic syndrome, and involvement of the nuclei in tegmentum of the brainstem explains gait instability, movement disorders, and oculomotor deficits.

## Immunology and Pathophysiology

The IgLON proteins are a family of cell adhesions molecules, containing five different proteins (Opiod-binding cell adhesion molecule (IgLON1), neurotrimin (IgLON2), limbic system-associated membrane protein (IgLON3), neuronal growth regulator 1 (IgLON4) and IgLON5). Structurally IgLONs are ~340 amino acids long and tied to the cell membrane. They possess three Ig-like domains. Through the first Ig-domain, it has recently been indicated that IgLON's can homo- and heterodimerize with high affinity and in a Ca^2+^ independent manner, thus making them able to interact across different cells and/or the synaptic cleft (23).

The complete function of the proteins and their interactions remain largely unclear. It is, however, known that the IgLON proteins play a role in neuronal development and synaptic formation (e.g., neurite sprouting and cell adhesion, astrocyte growth and limbic axonal differentiation), as well as cortical and hippocampal proliferation and synaptogenesis (2, 24–26). It has been suggested that IgLONs play a role in the evolution of brain anatomy and complex maturation, influencing cellular migration and brain-blood barrier integrity (27).

Polymorphisms in the IgLON1 and IgLON2 gene have been found to be associated with late onset Alzheimers disease (28), whereas IgLON3 has been linked to schizophrenia and depression (29). This is intriguing knowledge considering the phenotype of anti-IgLON5 disease with prominent symptoms such as dementia and behavioral symptoms, alongside tau depositions, and may further strengthen the hypothesis that damage to the IgLON5 protein is an underlying mechanism of the disease.

Patients with anti-IgLON5 disease display all four isotypes of immunoglobulin G (IgG) but predominantly IgG1 (94%) and IgG4 (92%), with a higher density of IgG4 than IgG1 in serum (Table 1).

The role of anti-IgLON5 antibodies in autoimmunity is an ongoing discussion. The main antibody target on IgLON5 is the Ig-like domain 2 and the IgG1 anti-IgLON5 antibody population has been shown to cause irreversible pathological internalization of IgLON5, supporting the immune mediated theory of the disease (2). However, majority of antibodies are IgG4, which have not been found to induce endocytosis of IgLON5 (2). It cannot be ruled out, that IgG4 antibodies may exert an effect on IgLON5 protein function or stability (as seen in other IgG4 antibody mediated AIE, such as LGI1 or Caspr2), this however remains to be further elucidated (2, 30).

Anti-IgLON5 disease differs from other AIEs because the assumed immunologic pathology is described in combination with neurodegeneration shown in neuropathological investigations. The main neuropathological findings are abnormal p-tau protein deposits (3-repeat and 4-repeat isoforms) in neurons, with no concurrent inflammatory infiltrates, beta-amyloid or alpha-synuclein deposits. Tau aggregates were predominantly found in the hypothalamus and tegmentum of the brainstem with a rostrocaudal gradient involving hippocampus, medulla oblongata, periaqueductal gray matter and the upper cervical cord (1, 3). Until now, only sparse glial and white matter involvement has been described. Microglia activation has been found in two cases (12, 31).

It is still unknown if antibodies are an epiphenomenon appearing secondary to the neurodegeneration or if the neurodegeneration is purely antibody mediated (32). But a combination of autoantibodies, a solid association to HLA haplotypes, and response to immunotherapy in some cases, supports the latter. A combination of HLA-DRB1^*^10:01 and HLA-DQB1^*^05:01, or HLA-DQB1^*^05:01 alone (in two cases) was found in 92% of cases of anti-IgLON5 disease, where HLA-type was tested (Table 1). The HLA-DRB1^*^10:01 allele is found in <2% of the general population and increased up to 36 times in patients with anti-IgLON5 disease (4).

## Diagnosis

Detection of anti-IgLON5 antibodies is crucial for diagnosis. Antibodies can be detected in both serum and CSF and are positive in 95 and 100% of cases, respectively (Table 1).

Routine CSF findings are normal in approximately half of cases. Elevated protein level is the most frequent finding, seen in 49% of cases. Pleocytosis is found in 24% of cases, and is mild with majority of cell count levels between 5 and 10 leukocytes/μL. OCBs are rarely present. Due to the neuropathology of the disease, tau, p-tau and beta-amyloid have been measured in CSF in some recent cases (10, 13, 18, 19, 31, 33). However, levels of p-tau were only increased in two out of seven cases (Table 1).

Evaluation of imaging findings revealed abnormal MRI findings in only 19% of patients. The most common finding was brainstem/cerebellar atrophy (45%). Of note, most of changes found on MRI are in regions shown affected by the tauopathy (hypothalamus, brainstem, hippocampus, cerebellum) (Table 2). Even though only a small number of patients had undergone brain FDG PET CT, abnormal findings were present in 50%, confirming the sensitivity of this modality in antibody mediated disease (34). All abnormal findings were hypermetabolism in regions that correlated with clinical symptoms and regions assumed affected by the tauopathy (basal ganglia, brainstem, cerebellum) (Table 2). Leptomeningeal microglial activation was visualized using translocater protein 18 kDa binding (TSPO) PET in one patient with a history of progressive tremor, sleep disturbances and gait impairment, and a tau-PET demonstrated cerebellar tau deposits in the same patient (10).

When performed, PSG was found abnormal in 95% of cases and symptoms included insomnia, low sleep efficiency, non-REM, and REM parasomnia with finalistic movements and sleep disordered breathing (stridor, respiratory failure, and OSA). Obstructive sleep apnea was present in 64% of cases (Supplementary Table 1).

Additional ancillary testing including electroencephalography, ENG, EMG, DAT SPECT, and whole body FDG-PET CT were mostly unremarkable or nonspecific.

## Treatment and Outcome

Overall, majority of patients diagnosed with anti-IgLON5 disease were treated with immunosuppressants (80%), but various combinations have been used. The most frequent used were cycles of IV corticosteroids (58%) in combination with IV immunoglobulins (IVIg−36%) and/or TPE (27%). The most commonly used second line treatments were Rituximab (22%) and Cyclophosphamide (12%), but other strategies such as Azathioprine and Mycophenolat Mofetil have also been used (5, 7, 10, 17) (Supplementary Table 1). Most of the cases reported have been treated with a combination of at least two different therapy strategies.

The 20 patients reported by Gaig et al., who received therapy, had a median disease duration of 34,5 months and only two of the 20 patients treated with immunotherapy had partial/transient effect of treatment. In the remaining 36 of reported cases, median disease duration was 12 months, and 24 cases received immunotherapy (in three cases, therapy was unknown). Seven of the 36 patients died.

**Figure 2:**
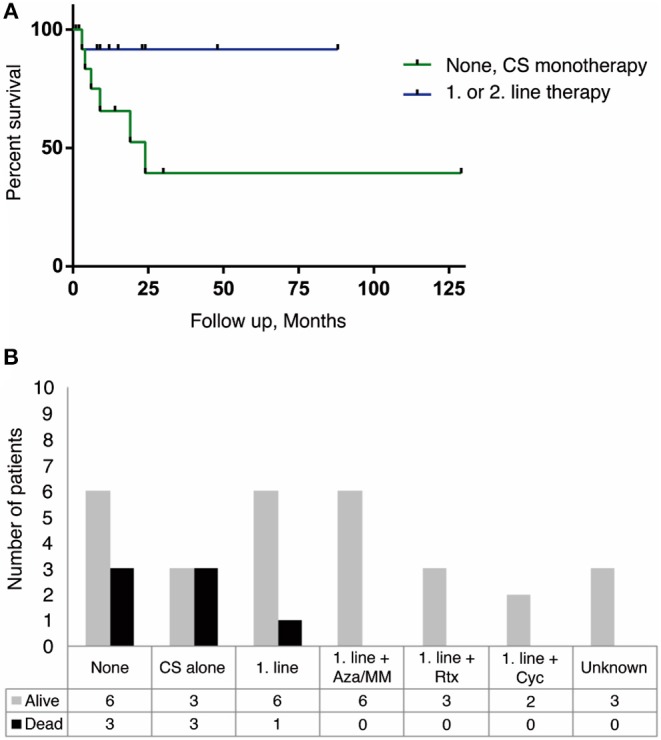
Treatment strategies and survival outcome in anti-IgLON5 disease. Patients receiving no therapy or CS monotherapy had a higher mortality than patients treated with a combination of 1. line therapy (CS+IVIg or TPE or IVIg/TPE alone) and a steroid sparing agent. Similarly, addition of 2. line therapy with Rituximab or cyclophosphamide improved survival. **(A)** Kaplan-Meier survival curve showing the difference in survival between no therapy and/or CS alone (green line) and 1. or 2. line therapy (blue line). It should be noted that follow-up time differed from case to case, resulting in a high number of censored data (black dot) within an already small population (*n* = 27, *p* = 0.064). **(B)** Outcome between different treatment strategies *n* = 36. CS, corticosteroids; IVIg, intravenous immunoglobulin; TPE, therapeutic plasma exchange; Aza, Azathioprine; MM, Mycophenolate Mofetil; Rtx, Rituximab; Cyc, Cyclophosphamide.

In cases where information about treatment was available, we found no clear correlation between disease duration and disability or death. However, patients who received no treatment or treatment with corticosteroid alone appear to have a higher mortality compared to patients receiving more potent immunotherapy. Six of the seven patients that died, received either IV steroids as monotherapy or no therapy (Figure 2). There was no clear correlation found between therapeutic strategy and degree of remaining disability in patients (reported and estimated mRS scores).

Overall, 20 out of 58 patients with definite anti-IgLON5 disease have been reported dead (34% mortality). The most common cause of death was sudden death (56%) followed by aspiration (44%). Death showed no clear correlation to treatment response, as even cases with partial response died suddenly (9, 14, 18) (Supplementary Table 1).

Symptomatic treatment with CPAP in patients with OSA improves respiratory symptoms, but has no convincing effect on parasomnias (20). In some patients with movement disorders (myoclonus, parkinsonism, and dystonia) antiepileptic, dopaminergic, and anti-hyperkinetic drugs were administered, but only with sparse effect on symptoms (7, 18, 19, 33).

## Conclusion

Anti-IgLON5 disease should be suspected in patients displaying sleep disorder characterized by insomnia, non-REM parasomnia, finalistic movements, and sleep disordered breathing in combination with bulbar symptoms, gait instability, involuntary movements, ocular abnormalities, neuropsychiatric symptoms, dysautonomia, and peripheral nervous system involvement. Antibodies against IgLON5 are crucial for diagnosis, and are present in serum and in almost all cases in CSF. HLA-DRB1^*^10:01 and HLA-DQB1^*^05:01 is strongly associated to presence of anti-IgLON5 antibodies. Brain FDG-PET CT is abnormal in 50% of cases, and could be more sensitive than MRI. Tau level in CSF, tau-PET or brain biopsy might support the diagnosis, but still needs further exploration. Aggressive immunotherapy seems to be crucial for outcome, as untreated patients or patients treated with steroid monotherapy appear to have a higher mortality. Further studies in larger cohorts with long-term follow up are needed.

## Data Availability Statement

All datasets generated for this study are included in the manuscript/Supplementary Files.

## Ethics Statement

Ethical review and approval was not required for the study on human participants in accordance with the local legislation and institutional requirements. The patients/participants provided their written informed consent to participate in this study. Written informed consent was obtained from the individual(s) for the publication of any potentially identifiable images or data included in this article.

## Author Contributions

MN and MB: design and draft of the manuscript, acquisition and interpretation of data, revised manuscript for intellectual content.

### Conflict of Interest

The authors declare that the research was conducted in the absence of any commercial or financial relationships that could be construed as a potential conflict of interest.
