# Supplementary material for: Anti-IgLON5 Disease: A Case With 11-Year Clinical Course and Review of the Literature
Source: Front Neurol. 2019 Oct 2;10:1056. doi: 10.3389/fneur.2019.01056 (PMC6783555; doi:10.3389/fneur.2019.01056)
Supplement: Supplementary file 1 [file Table_1.DOCX]

Supplementary table: Demographics, antibody status, symptoms, treatment and outcome in 58 cases with anti-IgLON5 encephalopathy

| **Patient no./sex/age/(ref)** | **Ab+** | **IgG class** | **HLA-DRB1*10:01**  **DQB1*05:01** | **Symptoms** | | | | | | | | | | | **Time to treatment** | | **Treatment** | | **Treatment response** | | **Outcome** | |
| --- | --- | --- | --- | --- | --- | --- | --- | --- | --- | --- | --- | --- | --- | --- | --- | --- | --- | --- | --- | --- | --- | --- |
|  |  |  |  | **Sleep disorder** | **OSA** | **Bulbar** | **Movement** | **Oculo-motor** | **Cerebellar** | **Gait** | **Psychiatric** | **Dyaautonomia** | **Peripheral** |  | |  | |  | |  | |  |
| 1/#/#/(4) | S, C | IgG 4 > IgG1 | + | + | # | + (#) | N/A | - | - | - | - | + (#) | - | N/A (# | | N/A (#) | | N/A (#) | | N/A (#) | |  |
| 2/#/#/(4) | S, C | IgG 4 > IgG1 | + | + | # | +(#) | N/A | - | - | - | - | +(#) | - | N/A (#) | | N/A (#) | | N/A (#) | | N/A (#) | |  |
| 3/#/#/(4) | S, C | IgG 4 < IgG1 | + | + | # | +(#) | + Chorea | - | - | + (#) | - | +(#) | - | N/A (#) | | N/A (#) | | N/A (#) | | N/A (#) | |  |
| 4/#/#/(4) | S, C | IgG 4 > IgG 1 > IgG2 |  | + | # | +(#) | N/A | + (#) | - | +(#) | - | - | - | N/A (#) | | N/A (#) | | N/A (#) | | N/A (#) | |  |
| 5/#/#/(4)) | S, C | N/A | + | + | # | +(#) | N/A | + (#) | - | +(#) | - | +(#) | - | N/A (#) | | N/A (#) | | N/A (#) | | N/A (#) | |  |
| 6/#/#/(4) | S, C | IgG 4 > IgG1 > IgG2 | N/A | + | # | +(#) | + Chorea | + (#) | - | +(#) | + Cognitive impairment (#) | +(#) | - | N/A (#) | | N/A (#) | | N/A (#) | | N/A (#) | |  |
| 7/#/#/(4) | S | IgG 4 > IgG1 | N/A | + | # | +(#) | + Chorea | + (#) | - | - | + Cognitive impairment (#) | +(#) | - | N/A (#) | | N/A (#) | | N/A (#) | | N/A (#) | |  |
| 8/#/#/(4) | S | IgG 4 > IgG1 | N/A | + | # | +(#) | + Chorea | + (#) | - | +(#) | - | - | - | N/A (#) | | N/A (#) | | N/A (#) | | N/A (#) | |  |
| 9/#/#/(4) | S, (C neg) | IgG 4 > IgG1 | neg. | + | # | +(#) | N/A | + (#) | - | +(#) | + Cognitive impairment (#) | +(#) | - | N/A (#) | | N/A (#) | | N/A (#) | | N/A (#) | |  |
| 10/#/#/(4) | S, C | IgG 4 > IgG1 | + | + | # | +(#) | + Chorea | + (#) | - | +(#) | + Cognitive impairment (#) | - | - | N/A (#) | | N/A (#) | | N/A (#) | | N/A (#) | |  |
| 11/#/#/(4) | S, | IgG4 = IgG1 > IgG2 | + | + | # | +(#) | N/A | - | - | +(#) | - | +(#) | - | N/A (#) | | N/A (#) | | N/A (#) | | N/A (#) | |  |
| 12/#/#/(4) | S, C | IgG4 > IgG1 > IgG2 | + | + | # | +(#) | N/A | + (#) | - | - | - | - | - | N/A (#) | | N/A (#) | | N/A (#) | | N/A (#) | |  |
| 13/#/#/(4) | S, C | IgG4 > IgG1 > IgG2 | N/A | + | # | +(#) | + Chorea | - | - | +(#) | - | +(#) | - | N/A (#) | | N/A (#) | | N/A (#) | | N/A (#) | |  |
| 14/#/#/(4) | S, C | IgG4 > IgG1 > IgG2 | neg. | + | # | +(#) | + Chorea | + (#) | - | +(#) | + Cognitive impairment (#) | - | - | N/A (#) | | N/A (#) | | N/A (#) | | N/A (#) | |  |
| 15/#/#/(4) | S, C | IgG4 = IgG1 > IgG2 | + | + | # | +(#) | N/A | - | - | +(#) | - | - | - | N/A (#) | | N/A (#) | | N/A (#) | | N/A (#) | |  |
| 16/#/#/(4) | S | IgG4 > IgG1 | + | + | # | +(#) | N/A | - | - | +(#) | + Cognitive impairment (#) | - | - | N/A (#) | | N/A (#) | | N/A (#) | | N/A (#) | |  |
| 17/#/#/(4) | S | IgG4 > IgG1 | N/A | + | # | +(#) | N/A | + (#) | - | +(#) | - | +(#) | - | N/A (#) | | N/A (#) | | N/A (#) | | N/A (#) | |  |
| 18/#/#/(4) | S, (C neg) | IgG1 | + | + | # | -(#) | N/A | + (#) | - | +(#) | + Cognitive impairment (#) | +(#) | - | N/A (#) | | N/A (#) | | N/A (#) | | N/A (#) | |  |
| 19/#/#/(4) | S | IgG4 > IgG1 | N/A | + | # | +(#) | N/A | + (#) | - | +(#) | + Cognitive impairment (#) | +(#) | - | N/A (#) | | N/A (#) | | N/A (#) | | N/A (#) | |  |
| 20/#/#/(4) | S, C | IgG 4<IgG1 >IgG2 | + | + | # | +(#) | N/A | + (#) | - | - | - | - | - | N/A (#) | | N/A (#) | | N/A (#) | | N/A (#) | |  |
| 21/#/#/(4) | S, C | IgG4 > IgG1 > IgG2 | + | + | # | -(#) | N/A | - | - | - | + Cognitive impairment (#) | +(#) | - | N/A (#) | | N/A (#) | | N/A (#) | | N/A (#) | |  |
| 22/#/#/(4) | S, C | IgG4 > IgG1 > IgG2 | + | + | # | +(#) | N/A | - | - | +(#) | - | +(#) | - | N/A (#) | | N/A (#) | | N/A (#) | | N/A (#) | |  |
| 23 / F / 69 () | S | # | N/A | - | - | - | + Parkinsonism, myoclonus, myorhytmia, dystonia, tremor | + Vertical gaze palsy | - | + | + Depression, memory) | - | - | N/A(#) | | none | | N/A | | mRS=6 | |  |
| 24 / M / 75 (5) | S, C | # | N/A | + | + | - | + parkinsonism, myoclonus | + Hypometric saccades | - | - | + Cognitive impairment, memory | + Urinary, bowel incontinence, anhidrosis | - | N/A(#) | | CS | | Improvement | | mRS =1 | |  |
| 25 / F / 62 (5) | S, C | # | N/A | + | + | + Dysphagia | + Chorea, dystonia | + Nystagmus | - | + Spastic | - | + Urinary incontinence | + UMN | N/A(#) | | CS + Aza | | Improvement | | mRS =4 | |  |
| 26 / F / 67 (5) | S | # | N/A | + | + | + Dysphagia | + Tongue movements, dystonia | - | - | - | + Hallucinations, delirium, memory | - | - | N/A(#) | | none | | N/A | | mRS=6 | |  |
| 27 / F / 72 (5) | S | # | N/A | - | - | + Dysphagia | + Parkinsonism | + Vertical gaze palsy, nystagmus | + Ataxia | + | + Cognitive impairment | - | - | N/A(#) | | none | | N/A | | mRS= 3 | |  |
| 28 / F / 70 (5) | S, C | # | N/A | + | + | + Resp. failure, stridor, laryngospam | +Parkinsonism, myoclonus, tremor | - | - | + | + Hallucinations, delirium, memory, depression | + Urinary incontinence | - | N/A(#) | | CS | | no response | | mRS=6 | |  |
| 29 / M / 63 (5) | S | # | N/A | + | + | + Dysphagia | - | Diplopia | - | - | + Anxiety | + Ortostatic hypotension, urinary hesitancy | - | N/A(#) | | none | | N/A | | mRS=1 | |  |
| 30 / M / 50 (5) | S | # | N/A | - | - | - | - | - | + Ataxia | + | - | + Anhidrosis | + LMN, cramps | N/A(#) | | none | | N/A | | mRS=2 | |  |
| 31 / F / 64 (5) | S | # | N/A | N/A | N/A | + Dysphagia | - | + (nystagmus) | + Ataxia | + | - | - | + LMN, polyneuropathy | N/A(#) | | CS, MM | | Improvement | | mRS=4 | |  |
| 32 / M / 61 (5) | S | # | N/A | + | + | + Dysphagia | - | - | + Ataxia | + | + Confusion, hallucinations | + Pollakisuria | + LMN, polyneuropathy | N/A(#) | | none | | N/A | | mRS=3 | |  |
| 33 / F / 66 (5) | C | # | N/A | + | + | + Dysphagia, resp. failure | +Parkinsonism | + Horisontal gaze palsy | - | + | - | - | + LMN, polyneuropathy | N/A(#) | | none | | N/A | | mRS=6 | |  |
| 34 / M / 59 (5) | S | # | N/A | + | + | - | - | + Ptosis | - |  | - | - | + LMN, cramps, fasciculations | N/A(#) | | none | | N/A | | mRS =1 | |  |
| 35 / M / 61 (5) | S | # | N/A | + | + | - | - | - | - | + Polyneuropathy | - | - | + LMN, paresthesias, polyneuropathy | N/A(#) | | CS, Cyc, + Aza | | Improvement | | mRS =1 | |  |
| 36 / F / 52 (5) | S | # | N/A | + | + | - | + Stiff person syndrome | - | - | + | - | - | - | N/A(#) | | CS, IVIg, MM, Rtx | | Improvement | | mRS = 1 | |  |
| 37 / F / 61 (5) | S, C | # | N/A | N/A | - | +laryngo-spasm | - | + Gaze palsy) | - | - | - | + Urinary retention | + LMN, fasciculations | N/A(#) | | N/A | | N/A | | mRS =3 | |  |
| 38 / M / 46 (5) | S | # | N/A | + | + | +Dysphagia | - | - | - | - | - | + GI dysmotility, anhidrosis | + LMN, cramps, fasciculations | N/A(#) | | IVIg, TPE | | Improvement | | mRS=2 | |  |
| 39 / F / 59 (5) | S | # | N/A | N/A | - | - | - | - | - | + | - | - | + Paresthesia, | N/A (#) | | N/A | | N/A | | mRS=3 | |  |
| 40 / M / 54 (5) | S, C | # | N/A | N/A | - | + Dysphagia | - | - | + Ataxia | + | - | - | - | N/A(#) | | CS | | no response | | mRS=2 | |  |
| 41 / M / 72 (5) | S, C | # | N/A | N/A | - | - | - | - | - |  | + Confusion |  | - | N/A(#) | | TPE | | Improvement | | mRS=1 | |  |
| 42 / F / 59 (5) | S, C | # | N/A | + | - | +Dysphagia | + Chorea | - | - | + | + Memory, disinhibition | + Urinary incontinence, constipation | - | N/A(#) | | CS, TPE | | Improvement | | mRS =2 | |  |
| 43 / F / 64 (7) | S, C | N/A | HLA DQB1*0501 | + | + | - | + Parkinsonism, chorea dystonia. | - | - | + Parkinsonism | + Cognitive impairment, memory | - | +Paresthesia (mouth) | 1 year | | IVIg, MM | | Improvement | | emRS=1 | |  |
| 44 / F / 77 (9) | S | N/A | N/A | + | N/A | +Dysphagia, resp. failure, vocal cord palsy | - | + Ptosis | - | - | - | - | + LMN, peripheral facial palsy | 2 years | | CS, TPE | | Improvement | | mRS=6 | |  |
| 45 / M / 45 (11) | S, C | N/A | + | + | + | +Dysphagia, Dysarthria, stridor | - | - | + Ataxia | + | + Confusion, depression | - | - | 1 year | | 1. CS+ IVIg, 2. TPE, IVIg, Cyc | | Improvement | | emRS=1 | |  |
| 46 / M / 61 (8) | S, C | IgG4 (S, C) | + | + | + | +Dysphagia, dysarthria | - | - | - | - | + Confusion | - | + UMN | 5 years | | IVIg, CS, MM | | Initial response | | mRS =4 | |  |
| 47 / F / 79 (13) | S, C | N/A | N/A | + | - | + Dysphagia, dysarthria | + Myokymia, myorrhytmia | - | - | - | - | - | - | 8 month | | N/A | | N/A | | N/A | |  |
| 48 / M / 49 (12) | S, C | N/A | + | + | + | +Dysphagia, dysarthria | + myorrhytmia, palatal tremor Intermittent trucal flexion movements at the hip every 2-4 seconds | - | - | - | - | - | - | 4 year | | TPE, IVIg, Rtx | | N/A | | N/A | |  |
| 49 / F / 75 (17) | S, C | IgG1 | + | - | - | - | + Dyskinesia lower limbs | - | - | - | + Hallucinations, apraxia | - | - | 0 years | | CS, TPE, Aza | | Improvement | | emRS=5 | |  |
| 50 / F / 70 (10) | S, C | N/A | N/A | + | - | - | + Postural and head tremor | + Abnormal saccades, nystagmus | + Ataxia, cerebellar eye movements | + Ataxia | + Depression | - | - | 1 year | | CS, Aza | | Improvement | | emRS=2-3 | |  |
| 51 / M / 58 (14) | S, C | N/A | N/A | + | - | +Dysphagia) | + Parkinsonism, abdominal dyskinesia | - | - |  | - | - | + LMN, Fasciculations, atrophy | 1 year | | CS | | Improvement | | mRS=6 | |  |
| 52 / F / 64 (6) | S; C | N/A | N/A | + | + | - | + involuntary movement of mouth, Parkinsonism | - | - | + Parkinsonism | - | - | - | 1 year and 8 months | | IVIg | | Improvement | | emRS=1-2 | |  |
| 53 / M / 73 (15) | S, C | N/A | N/A | + | - | Dysphagia, hoarseness. | - | - | - | + | + Confusion, Emotional lability | + Constipation, thermoregulation, urinary retention | - | 2 years | | none | | Improvement | | emRS=2-3 | |  |
| 54 / M / 57 (16) | S, C | N/A | + | + | - | +Dysphagia, resp. failure | - | - | - | - | + Abnormal behavior | - | + LMN, atrophy | 2 years | | CS, IVIg, TPE | | Initial response | | emRS=3-4 | |  |
| 55 / F / 71 (18) | S, C | N/A | N/A | - | - | + Dysphagia | - | - |  | + | + Dementia, depression | - | - | 6 months | | CS | | Improvement | | mRS=6 | |  |
| 56 / M / 62 (18) | S, C | N/A | N/A | + | - | + Dysarthria | + Myoclonus, dystonia | - | - | + | + Delusions, hallucinations | - | - | 1 month | | CS | | Initial respons | | N/A | |  |
| 57 / M / 56 (19) | S, C | N/A | HLA-DQB1*05:01 | + | + | + Dysartrhria | + Chorea, orofacial dyskinesia | + Abnormal saccades, nystagmus | - | - | + Cognitive impairment. | - | - | 6 month | | IVIg | | Improvement | | emRS=2 | |  |
| 58 / M / 61 (our case) | S, C | IgG4* | + | + | + | + Dysphagia, dysarthria, stridor, vocal cord palsy | + Dystonia | + Horisontal gaze palsy, ptosis | + Ataxia | + | + Cognitive impairment, confusion, disinhibition | - | + UMN, LMN, atrophy, fasciculations | 12 years | | CS, TPE, Rtx | | Improvement | | mRS=2 | |  |

# = data only available for total cohort and not in individual cases; - = not present; N/A = not available: *predominantly IgG4 based on cell based assay

Abbreviations: Aza = Azathioprine; Cyc = Cyclophosphamide; CS = corticosteroids;emRS = Estimated modified rankin scale; IVIg = intravenous immunoglobulin; LMN = Lower motor neuron; MM = Mycophenolate Mofetil; mRS = Modified rankin scale; Rtx = Rituximab; TPE = Therapeutic plasma exchange; UMN = Upper motor neuron
